# Outcome of retinopathy of prematurity patients following adoption of revised indications for treatment

**DOI:** 10.1186/1471-2415-8-23

**Published:** 2008-11-13

**Authors:** Aaron M Alme, Michael L Mulhern, Thomas W Hejkal, Jane L Meza, Fang Qiu, David D Ingvoldstad, Eyal Margalit

**Affiliations:** 1 University of Nebraska Medical Center, Department of Ophthalmology and Visual Sciences, Omaha, Nebraska, USA

## Abstract

**Background:**

The Early Treatment for Retinopathy of Prematurity study (ETROP), published in 2003, established new guidelines for treatment of retinopathy of prematurity (ROP) and demonstrated improved outcomes compared to previous guidelines. We examined outcomes before and after implementing the ETROP recommendations.

**Methods:**

A retrospective chart review was performed using records of infants who had laser ablations for ROP performed from January, 2000 through December, 2005. Data collected included date of birth; birth weight; estimated gestational age (EGA); grading of ROP; date of laser ablation; and outcome of laser surgery. Univariate association with threshold or prethreshold treatment (Pre-ETROP and Post-ETROP, respectively) were assessed using t-tests or Wilcoxon tests. Additional comparison between groups was performed using Fisher's exact tests.

**Results:**

581 patients were examined before and 464 after December 2003. Of these, 29/581 (5% – Pre-ETROP Group) and 53/464 (11% – Post-ETROP Group) patients advanced to criteria requiring laser treatment respectively (P = 0.0001). The average estimated gestational age (EGA) at birth was 26.3 and 25.2 weeks, with an average birth weight of 888 and 707 grams for Pre and Post-ETROP Groups, respectively. Stage 5 retinal detachment (RD) developed in 10.3% of eyes in the Pre-ETROP Group and 1.9% of eyes in the Post-ETROP Group (P = 0.02).

**Conclusion:**

After the ETROP guidelines were implemented, there was a decrease from 10.3% to 1.9% of eyes developing Stage 5 retinal detachment, despite this group having a lower average EGA and lower average birth weight. These results underscore the importance of adoption of the Revised Indications.

## Background

Retinopathy of prematurity (ROP) is a proliferative vascular retinopathy affecting infants of young gestational age and low birth weight. First described by Terry in 1942, ROP remains a leading cause of lifelong visual impairment among premature children in developed countries [1-11].

The multi-center trial of Cryotherapy for Retinopathy of Prematurity (CRYO-ROP) showed that 44.4% of eyes with a history of severe ROP had a visual acuity at age 10 years of 20/200 or worse. Of those children with visual acuities better than 20/200, only 45.4% had a visual acuity of 20/40 or better [2,3]. This prompted investigators to pursue more effective approaches to treatment.

In the CRYO-ROP study, peripheral retinal ablation were performed in eyes when the severity of ROP reached a point at which the risk of progression to RD was approximately 50%; this was termed *threshold ROP *[4-8,11]. Threshold ROP was defined as 5 contiguous or 8 non-contiguous clock hours of stage 3 ROP in zone I or II in the presence of plus disease. While some surgeons expressed concern regarding the treatment risks of earlier intervention, others strongly advocated earlier treatment despite the possibility that the ROP in many of these patients may regress spontaneously.

The National Eye Institute in 1999 sponsored a multicenter study of early treatment for ROP (ETROP study).[8] Eyes of infants were randomized to a) standard management based on CRYO-ROP recommendations, or b) early peripheral retinal ablation, if they developed prethreshold ROP. Prethreshold ROP was defined as:

▪ Zone I, any stage ROP that was less than threshold

▪ Zone II, Stage 2 ROP with plus disease

▪ Zone II, Stage 3 ROP without plus disease

▪ Zone II, Stage 3 ROP with plus disease but fewer than 5 contiguous or 8 cumulative clock hours of neovascularization [8].

The ETROP data, published in December 2003, demonstrated a benefit of earlier treatment compared with conventional management, with regard to both structural outcome and grated visual acuity [8]. In the ETROP study, all infants < 1251 g were screened. Initial screening is generally recommended for infants with a birth weight of < 1500 grams or with a gestational age of 30 weeks or less.

Our research aim was to compare outcomes of infants with ROP managed according to the CRYO-ROP guidelines to those managed under the ETROP guidelines. The demographics of treated patients were also evaluated.

## Methods

Internal Review Board approval was obtained for a retrospective study of preterm infants who were evaluated and/or underwent treatment for threshold or prethreshold retinopathy of prematurity from January 1, 2000, through December 31, 2005.

Using Current Procedural Terminology (CPT) and International Statistical Classification of Diseases and Related Health Care Problems, 9th Edition (ICD-9) coding, the patients were selected from the University of Nebraska Medical Center database.

Data was collected from four hospitals served by the University of Nebraska Retina Service, covering Nebraska's two largest cities. This encompasses a metropolitan region of approximately 750,000.

The main outcome measure was retinal detachment (RD). Data points collected include date of birth; birth weight; estimated gestational age (EGA) at birth; multiparity; EGA, zone, stage, and presence or absence of "plus" disease at the initial and each subsequent examination; scheduled follow-up examination dates; intervention date; laser spots placed per eye; follow-up examinations; RD, including staging; and complications, including late RD and cataract.

Univariate association with threshold or prethreshold treatment (Pre-ETROP and Post-ETROP, respectively) were assessed using t-tests or Wilcoxon tests for the following variables: EGA at birth, weight at birth, interval between birth and first examination, interval between birth and surgery, interval between first examination and surgery, and number of laser spots. Fisher's exact tests or χ2 tests were used to determine whether treatment was associated with RD, cataracts, amblyopia, as well as evaluating the extent of zone, stage, and plus disease when the decision was made to go for surgery.

For patients who developed RD, Fisher's exact tests were used to determine if threshold versus prethreshold treatment was associated with extent of zone, stage, and plus disease, as well as when the decision was made to go for surgery, grade of RD, and multiparity. Wilcoxon tests were used to determine if provider treatment was associated with the number of spots placed on laser surgery, age at birth, weight at birth, and interval between birth and surgery.

For all patients who received laser ablation treatment, as well as for patients who developed a RD, provider reliability was evaluated using Wilcoxon signed rank tests. This evaluated the difference between days scheduled between examinations and actual days elapsed between examinations for each interval between adjacent examinations.

In addition, patients were divided into two groups; one had more than 7 days between the previous examination and the examination that determined surgery, and the other group had 7 days or less between these examinations. Birth weight and EGA were compared between these two groups using t-test or Wilcoxon test; and extent of zone, stage, and plus disease were compared between these two groups, when a decision was made to go for surgery, using Fisher's exact tests or χ2 tests.

Finally, the average follow-up time for Pre-ETROP and Post-ETROP groups were evaluated using the Wilcoxon test.

## Results

Of the 1045 patients examined during the six year interval, 581 patients were examined prior to adoption of the ETROP guidelines in December 2003 (group A), with an additional 464 patients evaluated after adoption of the ETROP guidelines (Group B). Twenty nine of the 581 (5%) were treated with laser photocoagulation (Pre-ETROP Group), while 53 of the 464 patients (11.4%) evaluated after adoption of the ETROP guidelines received PRP (Post-ETROP Group).

The average EGA at birth of the Pre-ETROP patients was 26.3 weeks, while the average EGA at birth of Post-ETROP patients was significantly lower at 25.2 weeks (P = 0.0012) (Table 1). Post-ETROP treated infants also had a significantly lower birth weight, measuring 707 grams versus 888 grams in the Pre-ETROP group (P = 0.0007) (Table 1).

**Table 1:** Characteristics of Pre-ETROP and Post-ETROP Groups

	Pre-ETROP	Post-ETROP	
	N = 29	N = 53	
Characteristic	Mean (SD)	Mean (SD)	p-value
Age at birth, in weeks gestation	26.3 (1.7)	25.2 (1.38)	0.0012
Interval between birth and examination 1, in days	36 (9.54)	49.5 (18.76)	0.0004
Weight at birth, in grams	888 (257.79)	707 (194.79)	0.0007
Interval between birth and laser surgery, in days	73 (12.6)	80 (18.34)	0.09
Interval between examination 1 and laser surgery, in days	37 (13.72)	30 (18.91)	0.05
Number of laser spots, right eye	1595 (703.2)	1698 (499.4)	0.46
Number of laser spots, left eye	1686 (683.63)	1573 (491.32)	0.41
Multiparity	5	8	0.99

Table 1 demonstrates the characteristics of Pre-ETROP and Post-ETROP Groups treated with laser. Statistically significant differences include age at birth, weight at birth, and the interval between birth and first examination. Fewer days were needed between the first examination and laser surgery (P = 0.05). No significant difference was found in the number of laser spots placed, nor in the prevalence of multiparity.

The average interval from birth to laser treatment increased in the Post-ETROP group, from 73 days in the Pre-ETROP group to 80 days in the Post-ETROP group. This difference did not reach statistical significance (P = 0.09) (Table 1). However, the interval between the first screening examination and laser treatment was greater in the Pre-ETROP group (37 days) than in the Post-ETROP group (30 days) (P = 0.05) (Table 1).

The amount of plus disease in treated eyes was found to be significantly higher in the Post-ETROP group (46%) as compared to the Pre-ETROP group (10%) (P = 0.0001) (Table 2). There was no statistically significant difference between Pre-ETROP and Post-ETROP groups in relation to the follow-up findings of amblyopia or cataract. Multiparity was also not found to be a significant variable. Finally, there was no statistically significant difference in the number of laser spots placed per eye (Table 1).

**Table 2:** Outcomes of Pre-ETROP and Post-ETROP Groups following treatment with laser photocoagulation

	Pre-ETROP	Post-ETROP	
	N = 29, eyes = 58	N = 53, eyes = 106	
Outcome	Frequency of RD (%)	Frequency of RD (%)	p-value
Patients with any RD (retinal detachment)	5 (17.2)	8 (15.1)	0.99
Right eye	4 (13.8)	7 (13.2)	0.99
Left eye	4 (13.8)	6 (11.3)	0.74
Patients with Stage 5 RD	3 (10.3)	2 (3.8)	0.34
Eyes with Stage 5 RD	6 (10.3)	2 (1.9)	0.02
Cataracts	2 (6.9)	2 (3.8)	0.61
Amblyopia	2 (6.9)	0 (0)	0.12
Plus disease before surgery	10 (34.5)	46 (86.8)	< 0.0001

Evaluation of outcomes of Pre-ETROP and Post-ETROP patients treated with laser photocoagulation. Pre-ETROP and Post-ETROP Groups had 29 and 53 patients and 58 and 106 eyes treated with laser, respectively. A significant decrease was found when analyzing Post-ETROP eyes that developed Stage 5 RD (P = 0.02). This trend was true for the percentage of Post-ETROP patients as well, though it did not reach statistical significance. The amount of plus disease in the Post-ETROP group was found to be significantly higher. No difference was found between Pre- and Post-ETROP Groups with respect to cataracts, amblyopia, multiparity, or when comparing outcomes of right eye versus left eye.

**Figure 1:**
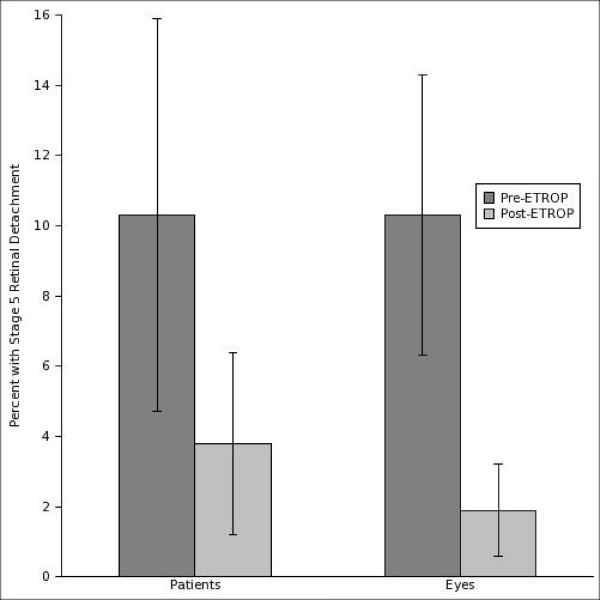
**Comparison of Stage 5 RD development between treatment groups.** Percentage of patients (*left*) and eyes (*right*) developing Stage 5 RD. There was a significantly lower incidence of Stage 5 RD in eyes of Post-ETROP vs. Pre-ETROP patients (*right*, P = 0.02). The incidence of Stage 5 RD was lower in Post-ETROP vs. Pre-ETROP patients (*left*), though this difference did not reach statistical significance (P = 0.34).

The percentage of treated *eyes *developing Stage 5 RD was significantly lower in the Post-ETROP group (2 of 106 eyes, 1.9%) as compared to the Pre-ETROP group (6 of 58 eyes, 10.3%) (P = 0.02). The percentage of *patients *treated with laser (as opposed to eyes) developing stage 5 RD was also lower in the Post-ETROP group (2 of 53 patients, 3.8%) than in the Pre-ETROP group (3 of 29 patients,10.3%), though this difference did not reach statistical significance (P = 0.34) (Figure 1 and Table 2). Cataracts were identified in 6.9% of Pre-ETROP patients (2 of 29), and 3.8% of Post-ETROP patients (2 of 53) (P = 0.61).

When examining serial records during the data collection phase, it was noted that some infants seemed to progress rapidly to threshold or pre-threshold disease and were followed more closely, while other infants developed clinically significant disease more gradually and were followed using standard follow-up dates based on the zone, stage, and plus component of the ROP. To evaluate whether the greater spacing in days between examinations was due to different patient characteristics, patients were divided into two groups; one had greater than 7 days between the previous examination and the examination that determined laser surgery, and the other group was 7 days or less between these examinations. Table 3 demonstrates no statistically significant difference in either gestational age at birth or birth weight between those two groups. A non-significant trend was noted for closer follow up for patients of younger gestational age at birth, and smaller birth weight.

**Table 3:** Timing of Examinations with relationship to EGA and birth weight

	Greater-than-7-day group (N = 26)			Less-or-equal-7-day group (N = 56)			p-value
	N	Mean (range)	SD	N	Mean (range)	SD	
Age at birth, in weeks gestation	26	25.92 (24–28)	1.47	55	25.44 (23–30)	1.64	0.14
Weight at birth	26	782.88 (396–1278)	231.22	54	767.39 (355–1347)	238.7	0.73

The examination that determines laser surgery (usually the same day or next day) may be related to age at birth or weight at birth. A trend was noted toward younger patients of less birth weight being followed more closely, but this did not reach statistical significance.

Analysis of the eyes treated with laser that developed RD was performed, comparing Pre-ETROP and Post-ETROP groups with respect to the zone, stage, and the presence of plus disease at the examination which determined laser surgery. As demonstrated in Table 4, there was no statistically significant difference found when comparing RD eyes by right eye versus left eye, by zone, or by stage. There was a statistically significant difference in the amount of plus disease in Post-ETROP versus Pre-ETROP patients (100% versus 40%, respectively) (P = 0.04). The Pre-ETROP Group had one twin (3.57%) and the Post-ETROP Group had four twins (8.16%). There is no statistical evidence of difference in the rate of twins between two groups (p-value = 0.65).

**Table 4:** Eye condition on final examination before laser surgery for patients who developed RD.

Characteristic	Pre-ETROP (N = 4)	Post-ETROP (N = 8)	
	Frequency (%)	Frequency (%)	p-value
Eye condition before surgery			
OD zone			0.99
1	1 (25)	2 (25)	
2	3 (75)	6 (75)	
3	0 (0)	0 (0)	
OS zone			0.99
1	1 (25)	2 (25)	
2	3 (75)	6 (75)	
3	0 (0)	0 (0)	
OD stage			0.41
1	1 (25)	0 (0)	
2	0 (0)	2 (25)	
3	3 (75)	6 (75)	
OS stage			
1	1 (25)	0 (0)	
2	0 (0)	3 (37.5)	
3	3 (75)	5 (62.5)	
Plus disease	2 (n = 5) (40)	8 (100)	0.04

Table 4 evaluates the pre-treatment findings in patients who developed a RD. Comparison between Pre- and Post-ETROP Groups demonstrate a statistically significant increase in the presence of plus disease in Post-ETROP patients as compared to Pre-ETROP patients. No other statistically significant differences were found.

To evaluate whether poor structural outcome could have resulted from failure to follow-up with serial examinations, the patients who developed RDs were evaluated with respect to the number of days scheduled between examinations versus the actual number of elapsed days between examinations. This was performed for both Pre- and Post-ETROP groups combined. Table 5 demonstrates the data analysis, which fails to find any aberration that reaches statistical significance.

**Table 5:** Scheduled versus actual days elapsed between examinations for patients treated with laser who developed a retinal detachment

		Number of days scheduled between examinations			Actual days elapsed between examinations			p-value
		N	Mean (range)	SD	N	Mean (range)	SD	
Pre-ETROP	Examination numbers							
	E1–E2	5	15.4 (14–21)	3.13	5	14.2 (7–22)	5.31	0.99
	E2–E3	5	10.2 (2–14)	5.5	3	16 (6–28)		0.99
	E3–E4	3	5 (1–7)	3.46	2	7.5 (7–8)		0.99
	E4–E5	2	0.5 (0–1)	0.71	0	--	--	--
Post-ETROP	E1–E2	7	9 (7–14)	3.42	7	8.57 (4–14)	4.08	0.72
	E2–E3	7	5.43 (1–14)	4.83	4	7.25 (5–11)	2.63	0.25
	E3–E4	3	7.33 (1–14)	6.51	2	11 (8–14)	4.24	0.99
	E4–E5	2	7 (7-7)	0	2	7.5 (2–13)	7.78	0.99

Pre- and Post-ETROP Groups are compared to determine whether there is any discrepancy between the number of days scheduled between examinations, and the actual number of days elapsed, in patients treated with laser who subsequently developed a RD. Data was analyzed to assess provider reliability, to determine whether an outlier exists, and if so, could it be a confounding variable in the difference in outcomes between Pre- and Post-ETROP Groups. No such outlier was found. (E1 = examination # 1, E2 = examination # 2, etc).

The average follow up time for Pre-ETROP and Post-ETROP groups was 492 days and 253 days respectively. Although there was a tendency for the Pre-ETROP subjects to be followed for a longer period of time, there was no statistically significant difference when comparing the two groups' follow-up time (P = 0.09) (Table 6).

**Table 6:** A Comparison of Folllow-up time between Pre-ETROP and Post-ETROP Groups.

Group	N	Mean	Std Dev	Std Error	Median	Minimum	Maximum
Pre-ETROP	28	491.75	599.47	113.29	304	1	2450
Post-ETROP	52	253.37	266.28	36.93	164	14	1135

Wilcoxon Two-Sample Test p-value: 0.09.

A Comparison of Follow-up time between Pre-ETROP and Post-ETROP groups. There is no statistical evidence of difference in the median of follow-up time between the two groups. While the Post-ETROP group showed shorter follow-up time than the Pre-ETROP Group, this was not statistically significant at the 0.05 level.

## Discussion

Our experience shows that after the ETROP guidelineswere implemented, there was a decrease from 11% to 1.9% of eyes developing Stage 5 RD, in spite of this group having a lower average EGA and lower average birth weight. The average EGA at birth trended downward, from 26.3 weeks in the Pre-ETROP group, to 25.2 weeks in the Post-ETROP group (P = 0.0012). This is not an unexpected trend, given the increasing survival of preterm infants under the care of our neonatal colleagues. These results underscore the importance of adoption of the ETROP guidelines for treatment.

The percentage of infants having treatment increased from 5% to 11.4%. Despite increased treatment, there was no appreciable offsetting detrimental effect from treating twice as many infants. Evaluation of our results showed no increase in patients developing cataracts under the ETROP guidelines; in fact, there is a non-statistically significant trend toward fewer cataracts in Post-ETROP as compared to Pre-ETROP patients (3.8% versus 6.9%, P = 0.61). Thus, it is reasonable to assume the benefit of increased treatment in patients who are treated according to the recommendations of the ETROP randomized trial outweighs any unrecognized detrimental effect.

Our data showed a statistically significant difference when examining the percentage of *eyes *that developed Stage 5 ROP. These results are consistent with results from the ETROP trial, which demonstrated a reduction in unfavorable structural outcomes from 15.6% to 9.1% (P < 0.001) [8].

There was a trend toward a longer interval between birth and laser treatment in Post-ETROP patients (Table 1), although it did not reach statistical significance. This most likely reflects the fact that infants in the Post-ETROP group had a lower EGA on average than those in the Pre-ETROP group. In contrast, there was a shorter interval between the first examination and laser treatment in Post-ETROP (30 days) versus Pre-ETROP patients (37 days). This is likely a consequence of the Early Treatment guidelines (prethreshold disease appearing earlier than threshold disease).

In addition, Post-ETROP patients had an increase in the amount of plus disease, as compared to Pre-ETROP patients. This may reflect the earlier gestational age at birth, the smaller birth weight, or simply the increased emphasis on the presence or absence of plus disease in the treatment algorithm of the ETROP guidelines protocol. The emphasis on presence or absence of plus disease in the ETROP guidelines may also contribute to the noted increase in plus disease.

Every effort was made to ensure that different providers did not unduly influence the outcome data. There is no difference in the number of laser spots placed between groups or with respect to the treated eye (Table 1). Further, there was no statistically significant difference found between providers in scheduled days versus actual elapsed days between examinations (Table 5), nor in the difference in the median follow-up time between the two groups (Table 6). These criteria are important when comparing patients treated by three different surgeons at our institution in the prethreshold versus threshold treatment arms.

Strengths of this study include careful evaluation of provider variability, and analysis of baseline characteristics of patients who developed poor structural outcome. The primary weakness of this study is related to the retrospective analysis. In addition, a larger sample size would further strengthen data that trended toward, but did not reach, statistical significance.

## Conclusion

In summary, we found a statistically significant difference in the outcome of ROP eyes treated under the ETROP criteria. Despite possible over-treatment, structural outcomes have improved, suggesting that the benefit of treating ROP in high-risk prethreshold disease outweighs the possible risks of over-treatment in a given high-risk population. Continuing investigation will allow for determination of even more specific high-risk factors for development of poor structural and visual outcome, narrowing the targeted high-risk population.

## Competing interests

The authors declare that they have no competing interests.

The authors have no proprietary interests in the materials discussed.

## Authors' contributions

AA participated in patient care, carried out the background research, generated the database, and drafted the manuscript and subsequent revisions. MM participated in the background research, generated the database, and developed the graphics. TH participated in patient care and revised the manuscript. JM provided statistical analysis. FQ provided statistical analysis. DI participated in patient care. EM participated in patient care, drafted the initial inquiry, participated in background research and database formation, and guided the revision and submission process.

## Pre-publication history

The pre-publication history for this paper can be accessed here:


